# High-frequency dynamic nuclear polarization in rotating solids

**DOI:** 10.1126/sciadv.aei6187

**Published:** 2026-09-16

**Authors:** Ravi Shankar Palani, Richard J. Temkin, Robert G. Griffin

**Affiliations:** ^1^ Department of Chemistry and Francis Bitter Magnet Laboratory, Massachusetts Institute of Technology, Cambridge, MA 02139, USA.; ^2^ Department of Physics and Plasma Science and Fusion Center, Massachusetts Institute of Technology, Cambridge, MA 02139, USA.

## Abstract

Nuclear magnetic resonance (NMR) spectroscopy is among the most powerful tools for determining molecular structure, yet its intrinsic sensitivity has long constrained what can be studied. Dynamic nuclear polarization (DNP) addresses this fundamental limitation by transferring the much larger polarization of electron spins to nearby nuclei, amplifying NMR signals by several orders of magnitude. When combined with magic angle spinning (MAS), a technique that averages anisotropic interactions in solid samples to yield high-resolution spectra, DNP transforms solid-state NMR into a practical tool for investigating complex biological assemblies, functional materials, and surfaces that are inaccessible to solution methods. Here, we review the current state and near-term future of high-field MAS DNP. We describe the principal continuous-wave polarization transfer mechanisms and their distinct dependencies on magnetic field strength, spinning frequency, and microwave power. We survey the development of polarizing agents, from early nitroxide biradicals to asymmetric and hetero-biradical designs that maintain efficiency at high magnetic fields above 18 T. We discuss the instrumentation that makes high-field DNP possible, particularly the gyrotron oscillator and emerging solid-state microwave sources. We discuss time-domain pulsed DNP as the solution to circumvent the field-scaling limitations of continuous-wave methods. Last, we highlight applications in structural biology, materials science, and surface chemistry where DNP-enhanced sensitivity has enabled measurements not otherwise feasible.

## BACKGROUND

Nuclear magnetic resonance (NMR) exploits a fundamental property of atomic nuclei: spin. When placed in a strong magnetic field, nuclei with nonzero spin—hydrogen (^1^H), carbon-13 (^13^C), and nitrogen-15 (^15^N)—precess at characteristic frequencies that depend both on the nuclei and on their local chemical environment. By detecting this precession, NMR spectroscopists can read out molecular structure, dynamics, and interactions with atomic resolution and without disturbing the sample. The technique underlies both modern structural biology and the magnetic resonance imaging (MRI) scanners used in clinical medicine.

In solution, molecules tumble rapidly and isotropically, averaging out the orientation-dependent (anisotropic) interactions between nuclear spins. This tumbling produces sharp spectral lines, analyzed by Bloembergen *et al*. (*1*), and has made solution NMR the workhorse for determining the structures of proteins, nucleic acids, and small molecules in their native liquid environment. Solid-state NMR, by contrast, deals with immobilized systems, membrane proteins, amyloid fibrils, catalytic surfaces, battery materials, and pharmaceutical formulations, where tumbling does not occur and anisotropic interactions broaden spectral lines. The remedy is magic angle spinning (MAS), where the sample is packed into a small rotor and spun at few tens of kilohertz about an axis tilted at precisely 54.74° relative to the magnetic field (*2*, *3*). This “magic angle” is the unique orientation at which the dominant orientation-dependent interactions average to zero provided the spinning frequency is comparable to or exceeds the magnitude of the interaction, recovering sharp lines and making high-resolution NMR of solids possible. Residual broadening from couplings (in hertz) larger than the available spinning frequency, notably strong ^1^H-^1^H dipolar couplings, is the principal reason ever-faster spinning is pursued. Alternative schemes such as magic-angle turning (*4*), which separates isotropic and anisotropic chemical shifts under slow spinning, and double rotation (*5*), which spins about two axes to additionally average second-order quadrupolar broadening, exist but are unnecessary for the spin-^1^/_2_ nuclei (^1^H, ^13^C, and ^15^N). MAS thereby opens solid-state NMR to the same kinds of structural and dynamic questions that solution NMR addresses, but for the vast range of systems that do not dissolve or that lose their functional form in solution.

However, a fundamental challenge remained: sensitivity. NMR is inherently a low-sensitivity technique. The thermal equilibrium polarization of nuclear spins is tiny, typically on the order of parts per million at room temperature and even at the highest available fields. This means that NMR experiments often require milligrams of material and hours of signal averaging and that many biologically and materially important systems simply cannot be studied without sensitivity enhancement. This limitation came into focus in the late 1980s, with the publication of experiments to measure internuclear distances, rotational resonance (*R*^2^) (*6*–*8*), rotary resonance recoupling (*R*^3^) (*9*), and rotational echo double resonance (REDOR) (*10*), and experiments to measure the molecular torsion angles ϕ, ψ, and χ (*11*–*13*). These papers established that MAS NMR experiments would permit the determination of high-resolution molecular structures. However, *R*^2^, *R*^3^, and REDOR, as well as subsequent developments in dipolar recoupling (*14*), rely on detection of ^13^C and ^15^N NMR signals and are therefore relatively low sensitivity experiments. At the time and in contrast, solution NMR was using ^1^H detection (*15*) to increase sensitivity by a factor of ∼8 to 24 for ^13^C and ∼30 for ^15^N (*16*), and this substantial improvement enabled solution NMR to be used to determine protein structures. Although ^1^H detection was, in principle, possible with MAS NMR, the spectral lines were broad because of the limited MAS spinning frequencies (ω_r_/2π ≤ 10 kHz) and low fields. Thus, in our recoupling experiments studying the structure of the photocycle intermediates of bacteriorhodopsin (bR) (*17*), the issue of sensitivity limited what we could learn and provided a clear rationale for the development of dynamic nuclear polarization (DNP). These (*18*, *19*) and, subsequently, many other experiments using DNP were successful, and it is now evolving to a widely applicable experimental technique.

In parallel to the necessity of improving sensitivity in MAS-DNP experiments were developments in MRI, where, again, the issue of sensitivity limits the information content of the images. This problem stimulated the idea of performing DNP on a frozen sample at very low temperatures, followed by melting, and injection into a subject for imaging (*20*). Today, we know this procedure as dissolution DNP, and it is now used clinically (*21*, *22*). A second parallel development, the signal amplification by reversible exchange (SABRE) (*23*) and SABRE SHEATH (*24*, *25*) experiments, uses highly polarized parahydrogen and a catalyst consisting of a metal-based polarization transfer agent, usually an iridium complex, to transfer polarization to a molecule of interest. Last, it is possible to produce highly polarized 129Xe via exchange with Rb in the gas phase, which can then be used in NMR and MRI experiments to improve sensitivity (*26*).

While these parallel developments have greatly expanded the scope of DNP, the focus of this review is on high-field MAS DNP, where the challenge of achieving efficient polarization transfer at magnetic fields relevant to modern NMR spectroscopy remains an active area of development.

## MAGIC ANGLE SPINNING DYNAMIC NUCLEAR POLARIZATION

DNP circumvents the sensitivity limitation by transferring polarization from electron spins, whose equilibrium polarization exceeds that of nuclei by the ratio of gyromagnetic ratios (γ_e_/γ_1H_ ≈ 660; γ_e_/γ_13C_ ≈ 2600), to the nuclei of interest. The sample is doped with a stable paramagnetic polarizing agent, cooled to ∼100 K, and irradiated with microwaves resonant with the electron spins, amplifying the NMR signal by two to three orders of magnitude. Because the electron line is broad and the transfer must compete with electron and nuclear relaxations, efficient continuous-wave (CW) transfer requires tens of watts of microwave power at subterahertz frequencies, hence the central role of the gyrotron (see Instrumentation). The CW-DNP mechanisms relevant to high-field MAS differ in the number of spins involved, the matching conditions that they require, and their dependence on magnetic field (Table 1).

**Table 1.** Comparison of the three CW MAS-DNP mechanisms (Overhauser, solid, and cross effects), summarizing spins involved, characteristic polarizing agent and EPR line shape, microwave matching condition, relative CW power requirement, field dependence, and representative enhancement ε. Frequencies ω_μw_, ω_0S_, and ω_0I_ (where S denotes electron spin and I denotes nuclear spin) and linewidths δ (homogeneous) and Δ (inhomogeneous) are defined as in the text.

Mechanisms/spin system	Polarizing agent/EPR line	Resonance (matching) condition	μw power	Field dependence	Representative enhancement (ε)
**Overhauser effect (OE)**, two spins (1 e^−^ + 1 n)	Narrow-line monoradical (BDPA, mixed valence)	ω_μw_ = ω_0S_	**Low** (allowed electron spin transition)	Increases with *B*_0_ for narrow-line agents; not universal	∼70 (BDPA/oTP) (*49*); ≈90 at 14.1 T (*43*)
**Solid effect (SE)**, two spins (1 e^−^ + 1 n)	Narrow-line radical (trityl, BDPA), δ < ω_0I_	ω_μw_ = ω_0S_ ± ω_0I_	**High** (forbidden ZQ/DQ transitions)	Decreases with *B*_0_; efficiency ∝ ${\left(\frac{\omega_{1e}}{\omega_{0I}}\right)}^{2}$	Up to ∼526 (CW, 9.4 T) (*57*); ∼500 (chirped, 3.4 T) (*56*)
**Cross effect (CE)**, three spins (2 e^−^ + 1 n)	Bis-nitroxide/hetero-biradical, δ < ω_0I_ < Δ	$\omega_{0S_{1}}-\omega_{0S_{2}}=\omega_{0I}$	**Moderate** (saturation creating polarization gradient)	Raw ε generally falls with *B*_0_ (no simple power law); optimized agents recover high net sensitivity at 14.1 to 21.1 T (*73*)	≈400 (AMUPol, 9.4 T); highest net sensitivity at 14.1 to 21.1 T with hetero-biradicals (*73*) [e.g., SNAPol-1 (*75*)] and cAsymPol-POK (*72*)

The idea of transferring the high polarization in the electron spin reservoir to nuclear spins was proposed by Overhauser (*27*) and confirmed shortly thereafter by Carver and Slichter (*28*). For the following ∼30 years, the primary application of DNP was the production of polarized targets for nuclear scattering experiments and the niche subject of magnetic ordering of nuclear spins (*29*). However, beginning in the 1980s, it was used in combination with MAS NMR experiments at modest field strengths (60 MHz/40 GHz for ^1^H and electron spins, respectively) by Wind *et al*. (*30*), Yannoni and co-workers (*31*, *32*), and Schaefer and co-workers (*33*, *34*) to enhance low field MAS spectra, primarily ^13^C of polymers. Concurrently, both solution and MAS NMR experiments were moving to higher magnetic fields (400 to 900 MHz) and the absence of suitable microwave sources at frequencies above 100 GHz limited the application of DNP. It was therefore clear that broad applicability of DNP would require microwave sources delivering 10 to 100 W of power at frequencies of ∼140 to 600 GHz, operating stably for the extended periods needed for multidimensional signal averaging. The only source that satisfied these requirements was the gyrotron (*35*), aka cyclotron resonance maser, and we constructed a system that functioned at 140 GHz/5 T (*36*) and achieved a factor of 10 enhancement of ^1^H (211 MHz) and 40 for ^13^C in a sample of polystyrene doped with 1,3-bis(diphenylene)-2-phenylallyl (BDPA) at room temperature (*37*). Subsequently, we reported experiments at low temperatures using nitroxides as polarizing agents and the enhancements increased to ε ≈ 180 (*38*), and we developed MAS probes that spin at ∼100 K (*39*). Bruker BioSpin commercialized this technology, and it now exists in about 70 different locations around the world operating at 400-, 600-, and 800-MHz ^1^H frequencies, with one installation at 900 MHz (263, 395, 527, and 593 GHz for *g* ≈ 2 electron spins).

In addition, low-temperature MAS probes were developed initially using 4- and 3.2-mm rotors that achieve ω_r_/2π ≈ 13 kHz at ∼100 K. The low-temperature linewidth in frozen samples has contributions both from a frozen distribution of conformations (inhomogeneous) and from homogeneous broadening. In systems where the homogeneous contribution is substantial, faster spinning (and higher field) attenuate it (see Applications of MAS-DNP experiments). This motivated the development of probes using 1.3- and 0.7-mm rotors that achieve ω_r_/2π ≈ 40 and 65 kHz, respectively, at 100 K with N_2_ gas as a driving medium. At these spinning frequencies, the ^1^H spectrum begins to narrow and ^1^H detection experiments become feasible.

**Fig. 1.**
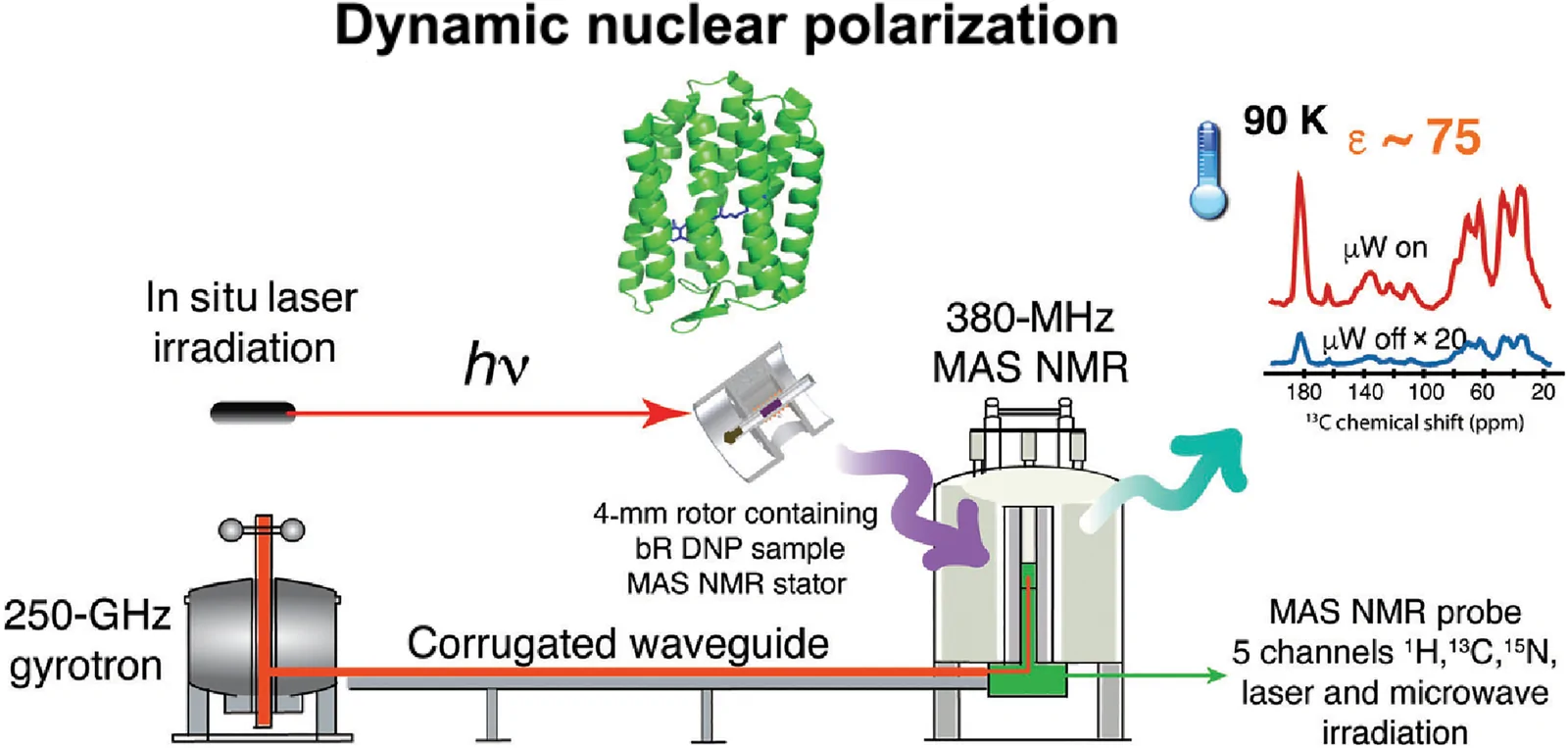
Schematic diagram of a typical CW-DNP MAS NMR spectrometer. The gyrotron generates ∼10 to 100 W of microwave power, which is transmitted to the DNP probe via a corrugated waveguide. The low-temperature NMR probe contains a corrugated waveguide with a miter bend at the complement of the magic angle to optimally irradiate the sample, which is contained in a sapphire or diamond rotor. Some probes have optic fibers for irradiating samples with laser light and all are triply tuned, usually to ^1^H, ^13^C, and ^15^N or ^17^O. This figure was adapted from Ni *et al.* (*19*). ppm, parts per million.

The schematic diagram shown in Fig. 1 illustrates the typical configuration of most of the MAS-DNP instruments now operating (*19*). It consists of a gyrotron oscillator connected to the low-temperature DNP probe by a corrugated waveguide. The MAS probe operates at 90 to 100 K, and the spinning frequency, ω_r_/2π, is determined by the speed of sound in N_2_ gas, which for 3.2-, 1.3-, and 0.7-mm-OD ZrO_2_ rotors is ∼13, 40, and 65 kHz, respectively. Custom-designed probes in our labs include optic fibers for photochemistry experiments on systems such as bR. At 9 T, the enhancements (ε) are ∼70 to 100 although, on model systems, ε = 420 to 526 is achieved.

## DNP MECHANISMS

In rotating solids, especially at high fields and during MAS, the dominant DNP mechanism is determined primarily by the line shape (the *g*-anisotropy) of the electron paramagnetic resonance (EPR) spectrum of the polarizing agent. In addition, several other factors including electron-nuclear couplings, relaxation rates and pathways, and even the sample spinning frequency contribute to the mechanism. CW-DNP mechanisms—notably, the Overhauser effect (OE), the solid effect (SE), and the cross effect (CE)—remain the workhorses high-field MAS-DNP experiments and exhibit distinct dependencies on magnetic field strengths, spinning frequency, and microwave power (Table 1) (*40*, *41*). A clear mechanistic understanding of these processes is essential not only for selecting optimal polarizing agents and experimental conditions but also for interpreting DNP-enhanced NMR spectra and for guiding the development of next-generation polarizing agents and pulsed-DNP technologies.

### Overhauser effect

The OE is the earliest DNP mechanism, predicted by Overhauser (*27*) and soon thereafter experimentally demonstrated by Carver and Slichter (*28*) in Li metal. The mechanism is traditionally associated with conducting solids and liquids, where stochastic motion drives electron-nuclear cross-relaxation. In the OE, microwave irradiation saturates an allowed electron spin transition, and polarization is transferred to nearby nuclei through zero-quantum (ZQ) and double-quantum (DQ) relaxation pathways. The sign and magnitude of the resulting nuclear polarization enhancement are determined by the imbalance between the ZQ and DQ cross-relaxation rates, yielding either positive or negative enhancements depending on which pathway dominates.

**Fig. 2.**
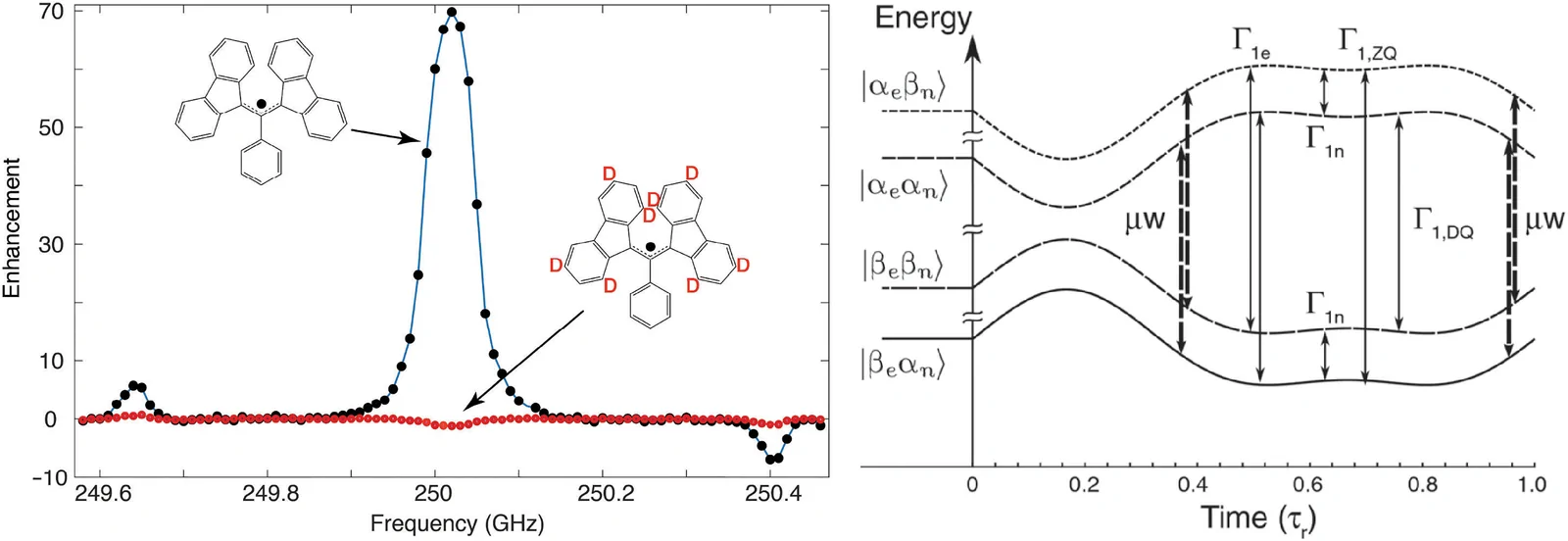
OE with BDPA in oTP. (**Left**) Zeeman frequency profile characteristic of an OE obtained from BDPA dispersed in *o*TP. The ε = 70 was obtained with a 160-mW output of AMC diode. Note that when *^2^H* is introduced into the α, γ positions of fluorenyl rings, we observe ε = −13, suggesting that e-^1^H hyperfine coupling at these positions is responsible for the OE. Because the power is low, the SE enhancements at ω_μw_ = ω_0S_ ± ω_0I_ are small, ε = ±7. (**Right**) Electron-nuclear energy levels of the BDPA radical as a function of orientation during one rotor cycle. The diagram illustrates the Γ_ZQ_ and Γ_DQ_ relaxation pathways in the two-spin system. Γ_e_ and Γ_n_ are the direct electronic and nuclear relaxation rates. The dashed arrows indicate the positions during the rotor cycle where the μw irradiation is on resonance for the SQ transitions. The EPR line in BDPA is narrow, and there is no level crossing. This figure was adapted with permission from Delage-Laurin *et al.* (*49*) (Copyright 2021 American Chemical Society) and Can *et al.* (*44*) with the permission of AIP Publishing.

For many years, the OE was considered irrelevant in insulating solids due to the absence of the translational motion required to modulate hyperfine interactions. However, this assumption was overturned by the observation by Corzilius and co-workers (*42*) of OE-DNP in insulating matrices doped with a sulfonated version of the narrow-line radical, BDPA. Unlike the SE and CE, which are discussed below and which additionally require specific matching conditions between the microwave and Larmor frequencies, the OE relies solely on the saturation of allowed electron transitions as shown in Fig. 2 (right). This enables efficient polarization transfer at relatively low microwave power. Crucially, for narrow-line agents such as BDPA the OE enhancement has been observed to increase with magnetic field (*43*, *44*), in contrast to the SE, whose efficiency scales unfavorably as $\omega_{0}^{-2}$. This favorable dependence is not, however, universal across OE systems: Methyl-driven OE in Blatter radicals (*45*, *46*) (discussed below) does not follow the same trend and only conduction-electron– and soliton-mediated OE are essentially field independent (*47*, *48*).

Recent mechanistic studies have revealed that, in insulating solids, the OE is mediated by intramolecular electron-nuclear interactions within the polarizing agent itself. In the case of BDPA, selectively deuterated radicals have demonstrated that strongly hyperfine-coupled protons on the fluorenyl moieties play a decisive role in determining the balance between ZQ and DQ cross-relaxation pathways (Fig. 2, left) (*49*). Protons with large (∼5 MHz) isotropic hyperfine couplings on the fluorenyl moiety promote ZQ-mediated transfer and positive nuclear enhancements, whereas weakly coupled protons on the phenyl moiety contribute to DQ-mediated transfer and consequently negative nuclear enhancements. The study led to optimization of the radical through selective deuteration of proton sites suppressing deleterious DQ pathways and has led to improved OE enhancements at high magnetic fields and moderate spinning frequencies, exceeding ε ≈ 90 at 14.1 T in MAS experiments (*43*).

The origin of the necessary time dependence in these rigid insulating solids remains a subject of active investigation. Pylaeva and co-workers (*50*–*52*) have proposed that the OE in BDPA-like radicals is driven by intramolecular charge-transfer and mixed-valence behavior. In this model, the radical undergoes rapid fluctuations between electronic states, effectively modulating the hyperfine interactions on a timescale comparable to the electron spin relaxation *T*_1e_. This hyperfine modulation provides the fluctuating field required for cross-relaxation even in a rigid lattice. This mechanistic insight suggested that the OE is not unique to BDPA but a general property that can be engineered into new classes of mixed-valence polarizing agents and was experimentally proven to be the case (*52*). Later, Perras *et al.* demonstrated methyl-driven OE-DNP using Blatter’s radicals, where rapid methyl rotations modulate electron-nuclear hyperfine couplings to protons, enabling efficient cross-relaxation for modest enhancements at high fields (∼9.4 and 18.8 T) (*45*, *46*). Consequently, the OE with narrow-line radicals such as BDPA and mixed-valence radicals represents a robust, low-power alternative for high-field DNP. There are other interpretations of the Zeeman field profiles illustrated in Fig. 2, which involve thermal mixing (TM). A discussion of these data is available in the perspective by Perras *et al.* (*53*)

### Solid effect

**Fig. 3.**
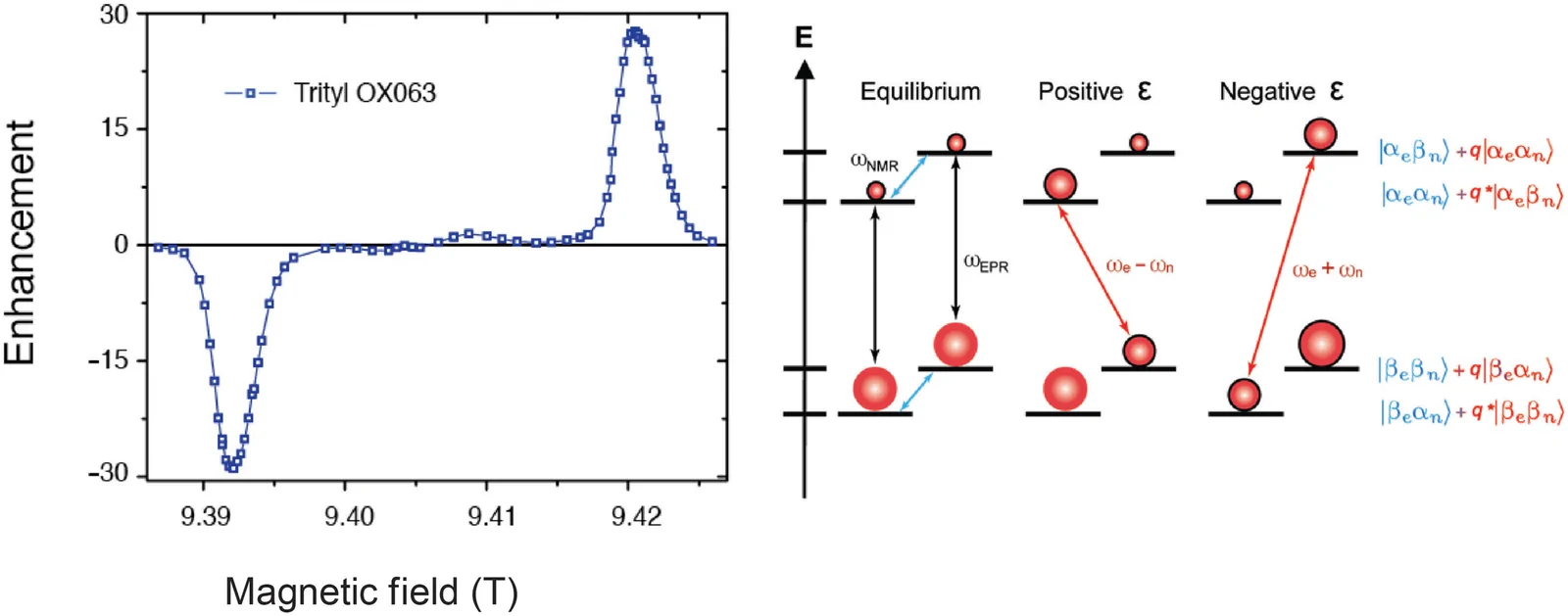
SE with trityl radical. (**Left**) SE Zeeman field profile recorded from a sample of trityl OX063 dispersed in d8-glycerol (60%)/D_2_O (30%)/H_2_O (10%) at 9.4 T (263 GHz/400 MHz) using a CW gyrotron. (**Right**) Energy levels associated with the SE transitions illustrating the state mixing that occurs mediated by $\left(S_{z}I_{\pm }\right)$ terms in the Hamiltonian. This figure was adapted from Can *et al.* (*44*) with the permission of AIP Publishing and with permission from Ni *et al.* (*40*). Copyright 2018 American Chemical Society.

Soon after the demonstration of the OE-DNP, the SE DNP was described for a coupled electron-nuclear spin system consisting of paramagnetically doped single crystals (*54*, *55*). In contrast to the OE, the SE polarization transfer occurs through the irradiation of nominally “forbidden” DQ or ZQ transitions $\left(\omega_{\mathrm{\mu w}}=\omega_{e}\pm \omega_{n}\right)$ as illustrated in Fig. 3 (left). These transitions become partially allowed due to the mixing of nuclear Zeeman states by the nonsecular part of the electron-nuclear hyperfine interaction $\left(S_{z}I_{\pm }\right)$ as illustrated in Fig. 3 (right). Depending on whether the ZQ or DQ transition is excited, the resulting nuclear polarization is either positive (DQ) or negative (ZQ). A Zeeman field profile for a SE experiment using trityl radical as a polarizing agent is shown in Fig. 3.

In the context of high-field MAS, the SE is the dominant mechanism when the polarizing agent is a narrow-line radical such as trityl or BDPA that satisfies the condition δ < ω_0I_, where δ is the homogeneous EPR linewidth and ω_0I_ is the nuclear Larmor frequency. While the SE provided the foundation for early DNP experiments, its efficiency, which scales as ${\left(\omega_{1e}/\omega_{0I}\right)}^{2}$, is determined by the state mixing required to drive the forbidden transitions. In contemporary MAS-DNP experiments, ω_0_ has increased and, for the most part, ω_1e_ remained constant, and, therefore, SE-DNP enhancements are lower at high magnetic fields. Because each transfer event is comparatively weak, the steady-state bulk enhancement is set by a competition between the rate at which the SE injects polarization and bulk nuclear spin-lattice relaxation (*T*_1n_), so that the SE is most advantageous for target nuclei whose longitudinal relaxation time *T*_1n_ is long relative to the polarization buildup time. This requirement is generic to the CW mechanisms but is hardest to satisfy for the SE at high field because of the ${\left(\omega_{1e}/\omega_{0I}\right)}^{2}$ dependency. Furthermore, in MAS experiments, the matching condition is only met transiently as the crystallite orientations evolve, leading to a series of discrete level anti-crossings. However, in a chirped pulse DNP experiment at 3.4 T, high pulsed power (300 W) was used increasing ω_1e_, and an enhancement of ∼500 was achieved (*56*). In a parallel development of CW-DNP, Emsley and co-workers (*57*) reported enhancements ε = 526 at 9.4 T (400 MHz) in a 1.3-mm rotor spinning at 20 kHz by using 60 W of microwave irradiation from a gyrotron source. These results demonstrate that the SE can be highly effective when the microwave excitation is sufficiently high.

### Cross effect

**Fig. 4.**
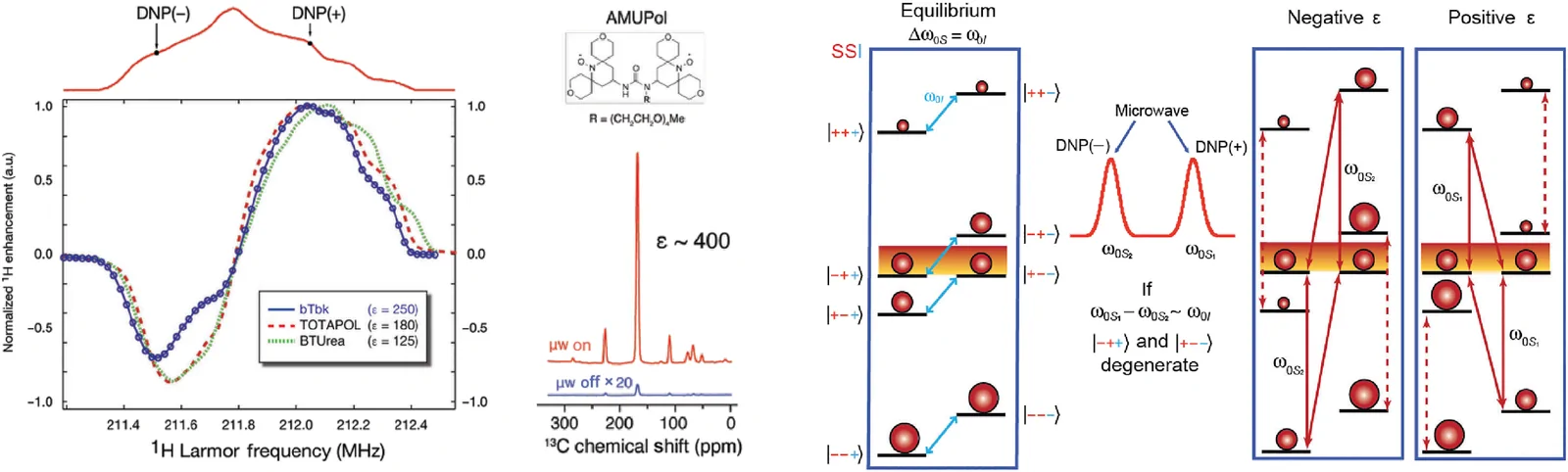
CE with biradical polarizing agents. (**Left**) Typical Zeeman field profiles recorded using bis-nitroxide biradical polarizing agents with *g*-anisotropies that satisfy the condition ($\delta <\omega_{0I}<\Delta$). Shown above the field profiles is the 140-GHz EPR spectrum, indicating the frequency positions of the maximum and minimum DNP enhancements. With bTbK, an early biradical, ε_max_ = 250. (**Center**) CE spectrum of ^13^C-urea using AMUPol as a polarizing agent yielding ε = 400. (**Right**) Energy level diagrams for a static three spin system such as a biradical with two electrons ($S_{2}$ and $S_{1}$) and a nuclear spin (I) with the size of the balls representing the nuclear spin populations of the states. The first part of the diagram shows the equilibrium Boltzmann populations. Upon irradiating the line at $\omega_{0S_{1}}$ (far right section), we excite the two transitions ∣−−−⟩→∣+−−⟩ and ∣−++⟩→∣+++⟩, and, because of the degeneracy of ∣−++⟩ and ∣+−−⟩, we also excite ∣−−−⟩→∣−++⟩ and ∣+−−⟩→∣+++⟩. Excitation of these transitions leads to an altered population distribution and a positive signal enhancement. Irradiation at $\omega_{0S_{2}}$ and similar arguments lead to a negative enhancement. This figure was adapted with permission from Ni *et al.* (*40*). Copyright 2018 American Chemical Society.

The CE, originally proposed by Kessenikh *et al*. (*58*–*60*), is now the most widely used mechanism to enhance signal intensities in high-field MAS DNP, particularly in biomolecular applications using bis-nitroxide biradicals as polarizing agents (*61*–*63*). In contrast to the SE, the CE is a three-spin process involving two coupled electrons and a nuclear spin, and, as illustrated in Fig. 4 (center), ε = 400 has been observed in model systems. It is exhibited by systems such as nitroxides where the inhomogeneous broadening, Δ, dominates the EPR line shape and exceeds the nuclear Larmor frequency of the coupled nuclear spin ($\delta <\omega_{0I}<\Delta$). The mechanism of the CE in the case of static samples is illustrated in Fig. 4 (right), where microwave irradiation is used to saturate one of the electron spin transitions, thereby creating a polarization gradient between the two electrons. A fraction of this polarization difference is subsequently transferred to the nuclear spin via three-spin flip-flop-flip transitions when the resonance condition, $\omega_{0S_{1}}-\omega_{0S_{2}}=\omega_{0I}$, is satisfied. This occurs when the difference between the resonance frequencies of the two electrons matches the nuclear Larmor frequency and is illustrated in Fig. 4 (far right). Note that irradiation at $\omega_{0S_{1}}$ saturates four levels and yields an increased positive polarization difference across the NMR transitions. Corresponding irradiation at $\omega_{0S_{2}}$ yields a negative enhancement as detailed in the figure caption.

**Fig. 5.**
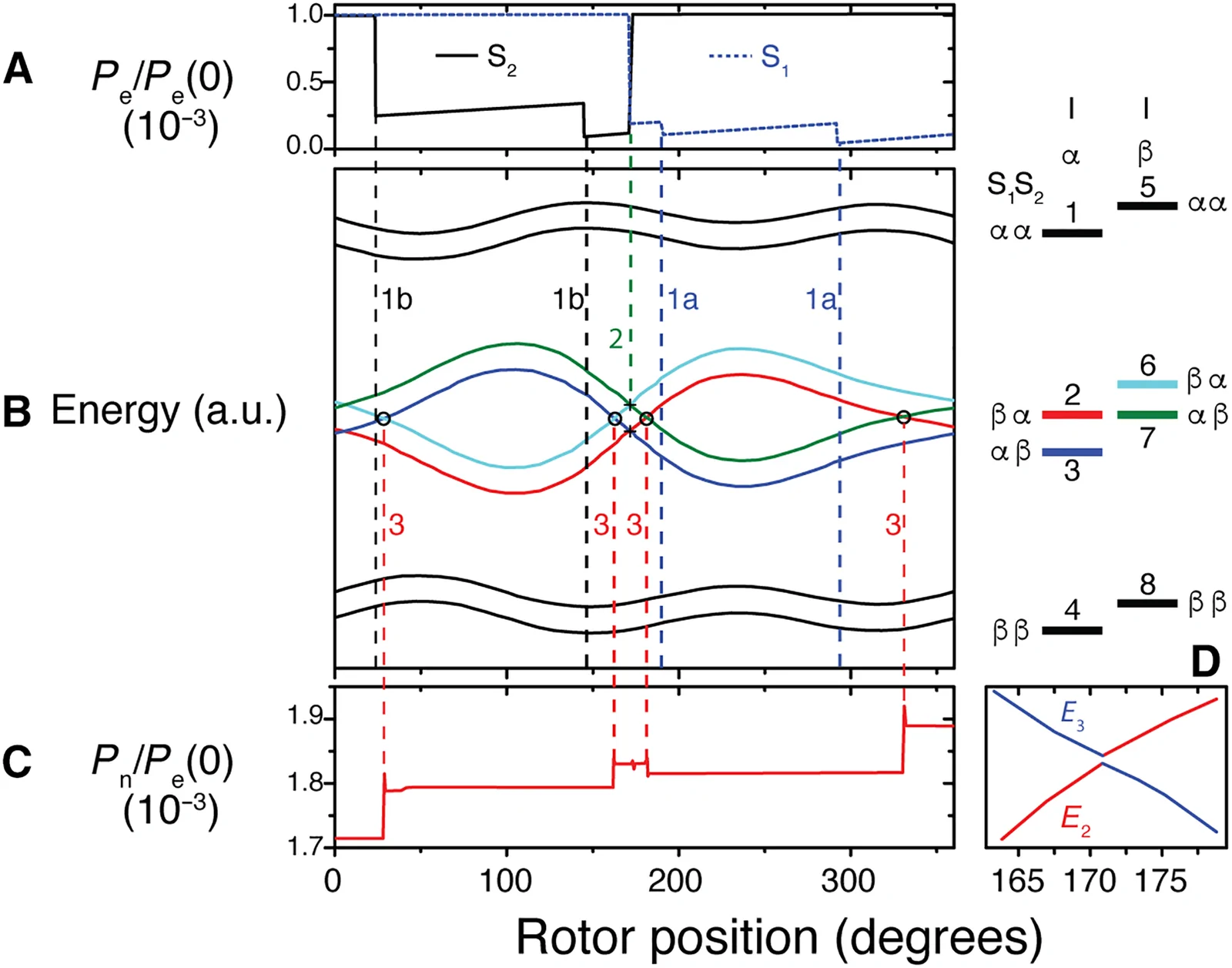
Cross-effect energy levels during a rotor cycle. Modulation of the electron energy levels in biradicals during sample rotation leading to electron nuclear polarization transfer. (**A**) Polarization of electrons during one rotor period. (**B**) The modulations of the energy levels during one rotor period due to anisotropic interactions including the *g*-anisotropy and the e-e dipolar coupling. (**C**) Nuclear polarization during one rotor period. (**D**) Electron-electron avoided level crossing. The dashed lines 1a and 1b indicate the flipping of the electron spins as the microwave frequency crosses one of the electron Larmor frequencies. The green dashed line 2 corresponds to the electron-electron avoided level crossing where the two electrons exchange their polarizations. The red dashed line 3 represents the three-spin avoided crossings at which DNP transfer occurs, as seen in the change of the nuclear polarization. [(A) to (D)] Reproduced with slight modifications from the simulations by Mentink-Vigier *et al.* (*161*) with permission from Elsevier.

While Fig. 4, which illustrates the CE mechanism for a static sample, provides a facile approach to understanding the CE, it is incomplete. Specifically, it does not account for the modulation of the energy levels that occurs because of the sample rotation and the orientation dependence of the *g*-tensor. As illustrated in Fig. 5, the energy levels of the central four levels periodically approach one another and undergo avoided level crossings that are mediated by perturbations from electron-electron dipolar couplings, electron-nuclear hyperfine interactions, and the applied microwave field. The efficiency of the DNP process depends critically on the adiabaticity of these crossings. Specifically, the passage through the crossing must be slow enough to allow spin populations to follow the adiabatic energy levels. Within a single rotor period, three distinct categories of avoided level crossing events occur: (i) an individual electron’s EPR frequency may cross the incident microwave frequency, resulting in saturation of the EPR transition; (ii) two EPR frequencies may cross each other, $\omega_{0S_{1}}=\omega_{0S_{2}}$; and (iii) the difference between two EPR frequencies may cross the nuclear Larmor frequency $\omega_{0S_{1}}-\omega_{0S_{2}}=\omega_{0I}$, driving the DNP enhancement. Maintaining high adiabaticity is particularly vital as it prevents the equilibration of electron spin populations and preserves the polarization gradient necessary for efficient CE-DNP.

The introduction of nitroxide biradicals such as TOTAPOL, bTbK, bTurea (*62*–*64*), and their improved versions such as AMUPol (*65*) and many others where the TEMPO moieties were oriented optimally with respect to each other (*66*) increased the efficiency of CE DNP severalfold. The development of polarizing agents for the CE has transitioned from empirical linker optimization to a rational design strategy based on advanced quantum mechanical simulations (*67*, *68*). One such insight is the importance of maximizing the norm of the difference between the two *g*-tensors, which ensures that a larger fraction of crystallite orientations satisfy the CE matching condition (*66*, *69*–*71*). This theoretical insight has driven the design of asymmetric bis-nitroxides, most notably the AsymPol family, which mimic the favorable spectral properties of hetero-biradicals by coupling chemically distinct nitroxide moieties (e.g., piperidine and pyrrolinoxyl rings) to prevent colinear *g*-tensor alignment and maintain a robust polarization gradient across the EPR line. For biophysical applications at high magnetic fields (>14.1 T) and higher MAS frequencies, this rational design philosophy has yielded highly efficient, water-soluble agents capable of hyperpolarizing proton-dense biomolecular assemblies (*72*). Prominent examples include cAsymPol-POK (*72*), which outperforms traditional agents like AMUPol by leveraging cyclohexyl substitutions on the pyrrolinoxyl ring to optimize electron relaxation properties, with structural rigidity provided by AsymPol tether (*72*, *73*). Furthermore, the field has expanded toward narrow-line/broadline hetero-biradicals including trityl-nitroxide radicals such as TEMtriPol (*74*), SNAPol and StaPol (*75*), as well as BDPA-nitroxide biradicals (*76*). PyrroTriPol, for example, exploits the narrow EPR line of its trityl component to achieve high enhancements (ε ≈ 120 at 9.4 T) while requiring two- to threefold less microwave power than bis-nitroxides (*77*). In addition, trityl-nitroxide radicals minimize depolarization and optimize CE efficiency at high fields while offering superior stability in reducing cellular environments, a crucial requisite for in-cell DNP studies. These next-generation radicals represent a notable leap toward applying high-field DNP for structural biology, ensuring high sensitivity even in challenging, biologically relevant conditions.

A final feature of the CE that occurs during sample rotation is “depolarization.” In the absence of microwave irradiation, the sample rotation leads to repeated passage through the CE matching condition and leads to a substantial loss of nuclear signal (*78*–*80*). Without an active microwave field that maintains an electron polarization gradient, these rotor-induced crossings facilitate a thermal equilibration between the nuclear and electron spin reservoirs. Because the electron spins relax quickly, they act as a sink, repeatedly drawing polarization away from the nuclei. This process, referred to as depolarization, causes the nuclear polarization to be reduced from its standard Boltzmann equilibrium by ∼40%, resulting in a lower detectable NMR signal than would be observed in a static sample (*66*, *69*). The final result is an overestimation of the DNP enhancement. A complete discussion of depolarization and other aspects of the CE DNP can be found in the book chapter by Hediger *et al.* (*81*), in the review articles by Corzilius and co-workers (*82*, *83*), and Gkoura and Equbal (*84*).

### Other CW-DNP mechanisms

Beyond the classical OE, SE, and CE mechanisms, additional CW-DNP mechanisms have been identified, during MAS experiments at high-fields, although these have not been used in applications of DNP. These include TM, which arises in systems with strongly coupled electron spin reservoirs where the homogeneous broadening of the EPR linewidth exceeds the nuclear Larmor frequency (*29*). Similar to the CE, TM involves electron-electron-nucleus three-spin transitions and typically requires higher concentrations of polarizing agents. Its contribution generally decreases at higher magnetic fields because the breadth of the nitroxide EPR spectrum grows with field while the electron-electron dipolar coupling stays constant, so the strong-coupling condition that TM requires becomes harder to satisfy.

Second, to explain dispersive DNP profiles frequently observed in narrow-line trityl radicals at high fields, a new mechanism, resonant mixing (RM), was recently proposed (*85*). Distinct from the SE (off-resonance irradiation) and CE/TM (multielectron processes), RM is effectively described as a single-electron, single-nucleus mixing process that emerges from state mixing under on-resonance microwave irradiation. Unlike the SE, which relies on forbidden transitions, RM is driven by allowed density-matrix evolution under weak microwave fields when the hyperfine coupling and microwave Rabi frequency interact constructively near the electron Larmor frequency.

Third, a truncated CE (*86*) has been observed in mixed-radical systems such as TEMPO-trityl, where one electron transition in a coupled pair is efficiently saturated while the partner electron remains only partially saturated or when spectral diffusion is inhibited. This produces an asymmetric CE frequency profile that can resemble an OE-like line shape despite arising from a CE-type three-spin process.

In practice, these CW-DNP mechanisms are not always mutually exclusive, and multiple polarization transfer pathways may operate simultaneously within a single sample. The dominant mechanism depends sensitively on parameters such as electron linewidth, radical concentration, magnetic field strength, MAS frequency, and microwave power. Recent studies on P1 centers in diamond powders (*87*–*90*) have provided clear examples of mixed-mechanism behavior, where features consistent with several CW-DNP mechanisms coexist under different experimental conditions.

## TIME DOMAIN DNP

While CW-DNP has enabled many high-field applications, its efficiency generally decreases with increasing magnetic field due to unfavorable scaling of state mixing and saturation conditions. In principle, time-domain or pulsed DNP offers an alternative in which polarization transfer is driven by coherent manipulation of electron-nuclear spin systems and can, in principle, circumvent the limitations of CW-DNP, enabling more efficient polarization transfer.

One of the earliest implementations of time-domain DNP is the integrated SE (ISE) (*91*, *92*), in which polarization transfer is driven by sweeping the resonance condition across the solid-effect matching transitions at $\omega_{0e}\pm \omega_{0n}$. In its original form, ISE was implemented by rapidly sweeping the static magnetic field through both solid-effect transitions, thereby integrating polarization transfer over the sweep. While such field sweeps are feasible at low magnetic fields, they become impractical at high fields, and, consequently, modern implementations keep the magnetic field constant and instead sweep the microwave frequency, an approach known as frequency-swept ISE (FS-ISE). In both cases, polarization transfer occurs through adiabatic passage at the electron-nuclear avoided crossings, where the sweep is sufficiently slow at the crossings to ensure adiabatic following of the coupled spin states yet fast overall to limit losses due to electron population relaxation (*T*_1e_).

Because full ISE traverses both solid-effect transitions, polarization transferred at the first crossing can be partially undone at the second if electron spin coherence is preserved between the two crossings. As a result, ISE is most effective when electron coherence is lost on a timescale comparable to or shorter than the sweep time interval between crossings, while population relaxation remains slow, i.e., *T*_2e_ ≤ *t* (between crossings) ≪ *T*_1e_.

Closely related chirped-pulse variants, developed recently, address this limitation by restricting the sweep bandwidth. In stretched SE (SSE) (*93*), the frequency sweep covers only one of the two solid-effect transitions, corresponding to approximately half the bandwidth used in FS-ISE, thereby avoiding coherent back-transfer and often improving transfer efficiency when electron coherence is longer lived. In adiabatic solid effect (ASE) (*56*, *94*), the sweep is further narrowed to remain localized around a single solid-effect transition, minimizing bandwidth and microwave power requirements while maintaining adiabaticity during the resonance passage. Together, ISE, SSE, and ASE represent distinct bandwidth-dependent regimes of the same underlying time-domain solid-effect mechanism, with ASE representing the narrowband limit that is particularly well suited to high-field and power-limited conditions, where maintaining adiabaticity over the full ISE bandwidth is impractical.

While ISE-based methods rely on adiabatic passage through solid-effect level crossings, an alternative class of pulsed DNP experiments exploits explicit spin locking in the rotating frame. Pulsed DNP experiments were initiated by Wenckebach and co-workers (*95*, *96*) by spin locking the electrons of the polarizing agent using a Rabi field that matches the nuclear Larmor frequency $\left(\omega_{1S}=\omega_{0I}\right).$ This experiment, nuclear orientation via electron spin locking (NOVEL), is therefore a rotating frame-lab frame polarization transfer analogous to a Hartmann-Hahn matching condition for heteronuclear cross-polarization and is the “gold standard” for pulsed DNP. It has been successfully performed at 9 and 34 GHz (*93*, *97*–*99*), but the implementation of NOVEL at higher magnetic fields remains challenging because of the large electron Rabi frequencies required. Accordingly, this constraint has stimulated the development of modified NOVEL schemes that either improve the efficiency of the transfer or relax its stringent power requirement. These include ramped-amplitude NOVEL (*98*), in which the spin-lock amplitude is swept through the matching condition to give a ∼1.6-fold improvement in transfer efficiency, and off-resonance NOVEL (*100*), which introduces a frequency offset Ω such that $\omega_{1e}^{2}+\Omega^{2}=\omega_{0n}^{2}$, so the matching condition can be met with a smaller Rabi field, achieving ∼70% of the on-resonance enhancement with only ∼11% of the microwave power and making it particularly useful in power-limited experimental conditions.

**Fig. 6.**
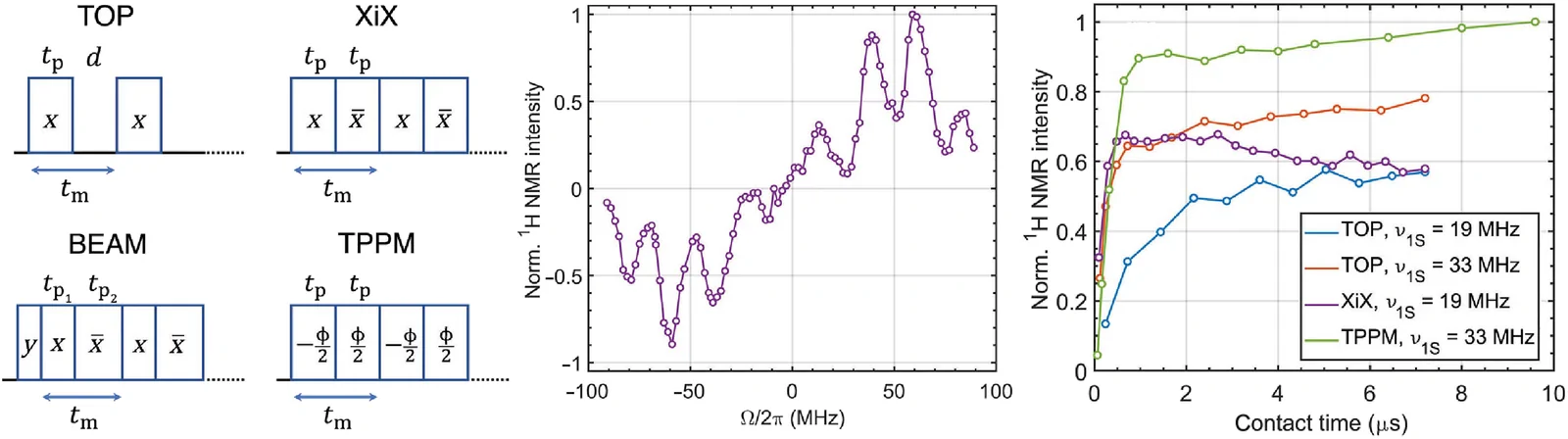
Pulsed DNP experiments at 34 GHz. (**Left**) Timing diagrams of the DNP pulse sequences TOP, XiX, BEAM, and TPPM that follow the matching condition $\left(n\omega_{0I}+k\omega_{m}+l\omega_{\text{eff}}\right)=0$. (**Center**) Typical microwave field profile of XiX-DNP sequence at $\omega_{1S}/2\pi$ = 18 MHz showing the typical oscillatory pattern observed with these sequences. (**Right**) Experimental transfer efficiency as a function of contact times for TOP ($\omega_{1S}/2\pi$ = 19 and 33 MHz), XiX ($\omega_{1S}/2\pi$ = 19 MHz), and TPPM-DNP ($\omega_{1S}/2\pi$ = 33 MHz). The polarizing agent used in the experiments was trityl radical at a concentration of ∼5 to 10 mM. This figure was adapted from Redrouthu *et al.* (*103*) under a CC BY license (https://creativecommons.org/licenses/by/4.0/deed.en).

In addition, there are now efforts to implement pulse experiments similar to those used in dipolar recoupling experiments. The first of these, which was performed at X-band frequencies, was time optimized pulsed DNP (TOP DNP) (*101*), and the timing diagram is shown in Fig. 6. It consists of a train of pulses separated by a defined delay, and the periodic modulation inhibits the averaging of the pseudosecular component of the hyperfine coupling by the nuclear Zeeman term, $\omega_{0n}\cdot {\hat{I}}_{z}$. This is analogous to the dipolar recoupling experiment in solid-state NMR, radiofrequency-driven recoupling, where the train of pulses inhibits the averaging of the dipolar coupling by the MAS. Subsequently, Redrouthu and Mathies (*102*) introduced the X-inverse-X (XiX) sequence, in which phase-alternated microwave pulses impose a symmetric modulation that suppresses noncommuting terms in the effective Hamiltonian, yielding ∼2-fold higher sensitivity than TOP DNP at comparable nutation frequencies. TPPM-DNP (*103*) extended this approach using phase-alternated pulse pairs analogous to TPPM decoupling in MAS NMR, providing a further ∼20% gain in enhancement and faster polarization buildup, although at the cost of higher electron nutation frequencies. More recently, Wili *et al.* (*104*) introduced BEAM-DNP, in which alternating the phase of the spin-lock pulses combines features of XiX and NOVEL to produce a broadband spin-lock condition that relaxes the strict matching requirement of NOVEL. An adiabatic variant (adiabatic BEAM-DNP), in which the duration of the second spin-lock pulse is swept through the matching condition, outperforms RA-NOVEL by ∼10% with added tolerance to microwave offsets and B_1_ inhomogeneity.

Beyond analytically designed pulse sequences, optimal-control–based approaches have emerged as a systematic framework for pulsed DNP, most notably from the groups of Nielsen (*105*–*107*) and Ernst (*108*). Here, microwave amplitude and phase waveforms are numerically optimized to maximize polarization transfer while incorporating experimental constraints such as finite power, bandwidth, and relaxation. Two complementary strategies have been pursued: state-to-state optimization, which maximizes transfer from electron polarization to nuclear ${\hat{I}}_{z}$, and periodic-sequence optimization, in which a compact pulse element is optimized and repeated to generate a well-defined effective Hamiltonian. The latter is particularly attractive as it yields robust, physically interpretable sequences that connect naturally to the average Hamiltonian descriptions of earlier pulse-train methods.

However, the practical implementation of pulsed DNP at high magnetic fields and under MAS conditions remains challenging, with most demonstrations to date limited to low fields and static samples. These limitations stem from the stringent microwave requirements of pulsed DNP, i.e., large electron Rabi frequency ($\omega_{1e}\gg 1/T_{2n}$), precise phase and amplitude control on nanosecond timescales, and hardware bandwidth constraints that are further compounded under MAS and at subterahertz frequencies. Nevertheless, recent advances in frequency-agile, phase-coherent microwave sources, improved coupling strategies, and high-performance rotor materials suggest that these constraints may be tractable, and time-domain DNP may yet emerge as a practical tool for efficient and selective polarization transfer in high-field (9.4 to 21 T) MAS NMR.

## PHOTOCHEMICALLY INDUCED DNP

Now, high-frequency microwave-driven DNP depends on the availability of a microwave source that is usually a gyrotron or high-powered klystron that is expensive. This has stimulated efforts to develop alternative approaches, and photochemically induced DNP was explored some time ago in photosynthetic reaction centers (*109*–*111*). More recently, a laser excited synthetic donor-chromophore-acceptor (DCA) molecule was used as a polarizing agent by De Biasi *et al.* (*112*–*114*) in successful experiments at 9.4 and 21.1 T. To date, the experiments yield ε ≈ 100 with the DCA molecule dispersed in an organic glass, perdeuterated ortho-terphenyl. A detailed analysis of the kinetics involved recently appeared (*115*). However, the long buildup times, a limitation shared with the OE and SE, remain the principal constraint, and extension to aqueous systems is complicated by the likelihood that the photoexcited chromophore reacts with labile protons. These remain open challenges despite the promise of the approach. Nevertheless, this is certainly an exciting addition to the repertoire of high magnetic field DNP experiments.

## INSTRUMENTATION

Efficient DNP at high magnetic fields critically depends on the availability of stable, high-power millimeter-wave radiation. As DNP experiments have moved to fields above 9 T and increasingly to 18.8 and 21.1 T (527 and 593 GHz, respectively), the technical demands on microwave sources have intensified, requiring tens of watts of CW output at precisely defined frequencies. Since its introduction at 140 GHz for DNP experiments on a 211-MHz NMR spectrometer (*36*–*38*), the gyrotron has become the dominant microwave source for high-field DNP and remains the workhorse technology for magnetic fields at 9 T and above (*116*). Its ability to reliably deliver high CW power for extended periods with good frequency stability has enabled many of the advances in high-field DNP over the past three decades. Thus, gyrotrons are commercially available that operate at 593 GHz, and we anticipate development of a 790-GHz system corresponding to 28.2 T (1.2 GHz for ^1^H), enabling DNP experiments at this and higher fields. The disadvantage of the gyrotron is that it is basically a fixed frequency oscillator and to record Zeeman field profiles necessary to understand DNP mechanisms or to optimize enhancements requires that the *B*_0_ field be swept, which could lead to the quench of the NMR magnet. In addition, a gyrotron requires a superconducting magnet that makes it an expensive addition to a spectrometer, and field-swept operation is likely impractical at the most cutting-edge magnetic fields.

**Fig. 7.**
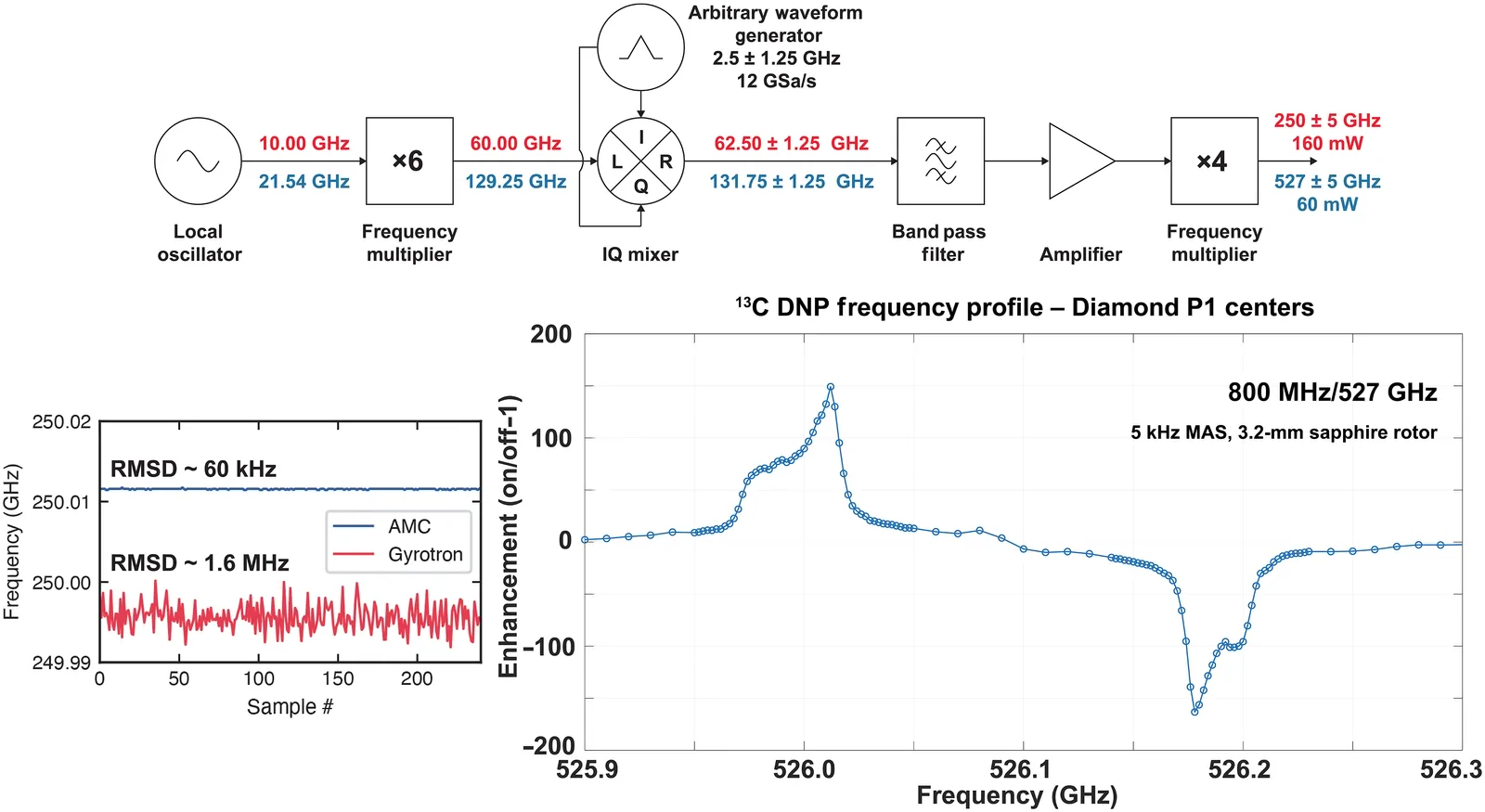
Frequency-swept DNP with a diode microwave source. (**Top**) Schematic of the solid-state microwave circuit used to generate microwave irradiation at 250 or 527 GHz for DNP experiments. (**Bottom**) Left: Frequency stability of the solid-state AMC diode source compared with a 250-GHz gyrotron demonstrating the frequency of the diode to be much less noisy (*117*). Right: ^13^C DNP frequency profile recorded of a P1 center–containing microdiamond powder sample, spinning at 5-kHz MAS in a 3.2-mm sapphire rotor, recorded at 527 GHz (800-MHz ^1^H frequency) using 60-mW solid-state microwave power. The 2-MHz step size, enabled by the AWG-integrated diode source, resolves fine structure in the profile that is inaccessible when sweeping the magnetic field, yielding enhancements of ε ≈ +149/−163.

In the last few years, there has been considerable progress in the development of solid-state diode sources, and we are operating two at 250 and 527 GHz producing ∼160 and ∼60 mW of output power, respectively (*117*). In addition, high-frequency arbitrary waveform generators (AWG) are now available, so that it is possible to mix the output of the diode with the AWG, which can be programmed to produce frequency, amplitude, and phase modulation of the microwave excitation. Similar systems are installed in several labs now at fields up to 9 T (*118*, *119*), and our lab recently implemented it at 18.8 T. A schematic of our circuit at 250 and 527 GHz is shown in Fig. 7 (top), where we also compare the spectral purity (bottom left) of the outputs of the diode with our homebuilt 250-GHz gyrotron (*120*–*122*). The 160-mW output was used in acquiring the frequency profile of the BDPA/*o*TP in Fig. 2, where we observed ε = 70. The diode source at 527 GHz/800 MHz and some of our initial results are illustrated in Fig. 7 (bottom right) where we show a ^13^C Zeeman frequency profile of a sample of P1 diamond with ε = +149/−163 obtained with 60 mW. Note the step size in this figure is ∼2 MHz in the regions of high enhancements so we can discern details in the frequency profiles that are difficult to distinguish when sweeping the magnetic field. In addition, we are not vaporizing liquid helium when sweeping the frequency. Last, traveling wave tube amplifiers are now operating at >200 GHz (*123*, *124*), and amplifiers operating in the 0.64- to 1.03-THz frequency range were demonstrated (*125*). Thus, it appears likely the instrumentation to perform serious time domain DNP experiments at high frequencies (>250 GHz) will soon be available.

Operating temperature is set by the sample-cooling hardware, and there is growing interest in pushing below the conventional ∼90 to 100 K. Lower temperatures bring substantial gains: Electron and nuclear relaxation slow, the equilibrium electron polarization increases, and depolarization losses are reduced, so that a given polarizing agent becomes markedly more efficient. Thurber and Tycko (*78*, *79*) showed that low-temperature operation is particularly advantageous for the level-crossing–driven CE. Helium-spinning MAS systems have been developed and demonstrated in the labs of Matsuki, Fujiwara and co-workers (*119*, *126*, *127*), De Paëpe and co-workers (*128*, *129*), and Thurber and Tycko (*130*). Realizing these gains also depends on rotor and stator design. Diamond rotors are particularly attractive in this context for their exceptional mechanical strength that permits faster spinning, including with helium as the bearing and drive gas, than ZrO_2_, their low microwave absorption that allows more of the incident power to reach the sample, and their high thermal conductivity that dissipates the heat from microwave and RF irradiation and from fast spinning, aiding temperature stability.

## APPLICATIONS OF MAS-DNP EXPERIMENTS

**Fig. 8.**
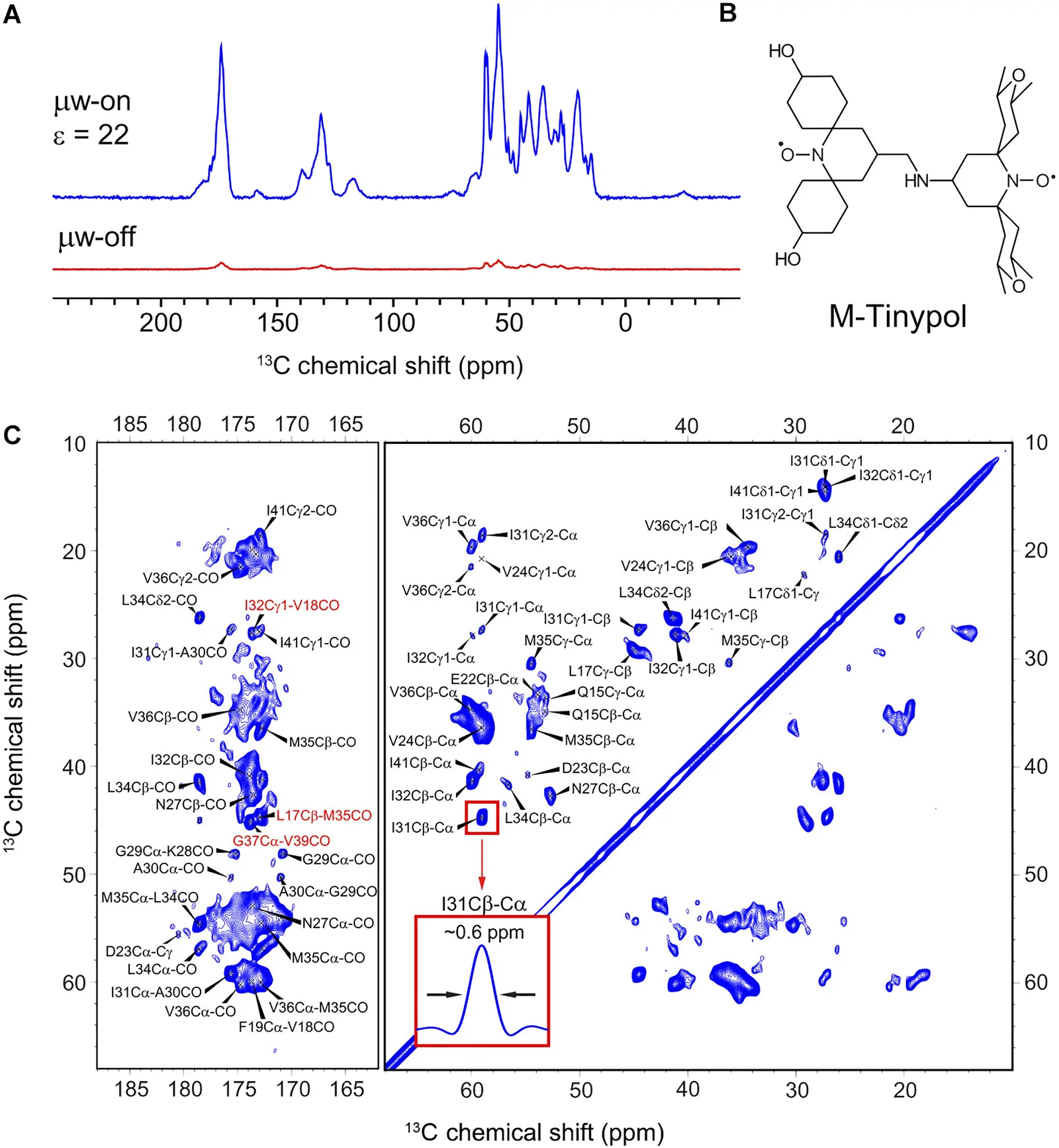
Biomolecular MAS DNP of Aβ_1-42_. Spectra were recorded at 800 MHz/527 GHz at 100 K. (**A**) ^13^C cross-polarization spectra recorded with (blue, μw-on) and without (red, μw-off) microwave irradiation, yielding a DNP enhancement of ε = 22. (**B**) Chemical structure of M-Tinypol, the bis-nitroxide biradical polarizing agent used in these experiments. (**C**) Two-dimensional ^13^C-^13^C correlation spectra. The protein was labeled with 1,3-^13^C_2_-glycerol and 2-^13^C_1_-glycerol. The linewidths and resolution at 100 K were identical to that observed at room temperature. The figure was adapted from Bahri *et al.* (*136*).

In the above sections, we discussed current and some possible future methods to perform MAS-DNP experiments. The development of these methods was stimulated by the desire to use MAS for applications that were not otherwise possible without increased sensitivity. The example of studies of photocycle intermediates of bR mentioned in Background was one of the initial applications to membrane proteins (*18*, *131*). Since then, MAS DNP has been applied across a broad range of biomolecular systems—other membrane proteins (*132*, *133*) and microbial rhodopsins (*134*, *135*), amyloid fibrils (*136*, *137*), protein assemblies and complexes (*138*–*140*), nucleic acids (*141*–*143*), and, increasingly, proteins within intact cells and native cellular environments (*144*–*146*)—with comprehensive surveys available elsewhere (*14*, *83*). The factor that has limited these studies is that the resolution decreases at low temperatures presumably because a distribution of conformations is frozen in place at ∼100 K, yielding a distribution of chemical shifts (inhomogeneous broadening). While this may be the case for some systems, a few recent experiments suggest other explanations. For example, a study of the type III secretion needles demonstrated that MAS spectra at 100 K are homogeneously broadened, and the linewidths narrow by observing spectra at 800 MHz rather than 600 MHz (*147*). A second demonstration of this behavior is illustrated in Fig. 8, which shows a MAS-DNP spectrum of Aβ_1-42_ recorded at ω_r_/2π = 40 kHz and 800 MHz (*136*). In this case, the resolution is identical in spectra recorded at 300 and 100 K, whereas spectra recorded at 100 K and 600 MHz at ω_r_/2π = 12.5 kHz are substantially broader (*148*, *149*). A third case where excellent resolution was reported involved nucleic acids where Hoogsteen base pairs in nucleic acid samples were observed because of the resolution and the improved sensitivity (*141*, *142*). These studies establish that the broadening observed at low temperatures in some samples is homogeneous rather than inhomogeneous in origin. Furthermore, methods to attenuate homogeneous broadening involve spinning rapidly and operating at high fields. Beyond the attenuation of homogeneous broadening, higher field confers a second, independent benefit: As in conventional NMR, the chemical-shift dispersion increases linearly with *B*_0_, separating otherwise overlapping resonances and extending MAS DNP to larger and more complex assemblies. Together with faster spinning, this motivates the development of DNP instrumentation operating at $\omega_{r}/2\pi$ > 40 kHz and at 18.8 T and higher fields.

In addition, some of the most successful applications of MAS DNP are to studies of surfaces (*150*), and these results stimulated investigations of other problems in materials science. Studies of materials often use MAS NMR, and, while MAS is an effective tool for elucidating structures, its low sensitivity is often problematic. DNP has therefore become the method of choice to enhance signal intensities. A further strength in this context is that the enhancement is often sufficient to permit measurements at natural isotopic abundance, so that surface sites, dopants, and dilute functional groups that cannot be selectively labeled are characterized directly (*151*–*153*). Working at natural abundance can be advantageous in other ways than overcoming difficulty in labeling: The dilution of ^13^C suppresses dipolar truncation and enables long-range ^13^C-^13^C correlations and, for organic solids, complete de novo chemical-shift assignments and structure determination that are inaccessible in uniformly labeled samples (*154*–*156*). This capability extended recently to artifact-free correlations at ultralow temperatures (*129*). Areas that have been of particular interest include metal-organic frameworks, perovskites, heterogeneous catalytic systems (*157*), functionalized silica, and polymers. Three excellent reviews of this area are available (*158*–*160*).

## CONCLUSION

DNP began as an interesting intellectual exercise in spin physics applicable to a limited set of conducting metallic samples. In the following three decades, its application expanded to include insulating materials such as paramagnetically doped single crystals and, subsequently, polymers. However, the primary application was limited to the production of polarized targets for nuclear scattering experiments and the study of magnetic ordering at ultralow temperatures, important but narrow applications, all confined to low magnetic fields. The transformation of DNP into applications for chemistry began in the 1980s, but broad application came with the introduction of high-frequency gyrotrons in the 1990s to perform the experiments in the high fields used in contemporary high-resolution NMR. This enabled the experiments on membrane proteins, amyloid fibrils, catalytic surfaces, and materials that have since established MAS DNP as an indispensable technique, now practiced in ∼70 laboratories worldwide.

The frontier that lies immediately ahead is the extension of DNP to higher magnetic fields and faster spinning frequencies, where both resolution and sensitivity stand to improve substantially. Achieving this will require advances on several fronts simultaneously, including polarizing agents engineered for efficient CE, SE, and OE transfers at fields above 18.8 T. In addition, microwave instrumentation such as frequency-swept solid-state diode sources and traveling-wave tube amplifiers or gyroamplifiers, capable of delivering coherent, phase-controlled radiation above 500 GHz with sufficient power are required. Third, time-domain polarization transfer methods that exploit the full versatility of pulsed microwave excitation are under development. These could transform DNP in the same way that pulsed experiments transformed the field of NMR a half-century ago. The photochemically induced DNP demonstrated recently at 9.4 and 21.1 T suggests that microwave-free routes to high-field hyperpolarization may also become practical. Each of these directions, pursued individually, represents a meaningful advance; pursued together, they suggest a trajectory in which the sensitivity limitations that have historically constrained solid-state NMR become progressively less binding. As sensitivity continues to rise, the technique reaches ever more dilute and chemically unmodified systems—increasingly at natural isotopic abundance in materials and surface science and, in favorable cases, within native cellular environments—extending solid-state NMR to biological, chemical, and materials problems previously beyond reach.
